# CircRNA_30032 promotes renal fibrosis in UUO model mice via miRNA-96-5p/HBEGF/KRAS axis

**DOI:** 10.18632/aging.202947

**Published:** 2021-05-11

**Authors:** Lei Yi, Kai Ai, Huiling Li, Shuangfa Qiu, Yijian Li, Yinhuai Wang, Xiaozhou Li, Peilin Zheng, Junxiang Chen, Dengke Wu, Xudong Xiang, Xiangping Chai, Yunchang Yuan, Dongshan Zhang

**Affiliations:** 1 Department of Emergency Medicine, Second Xiangya Hospital, Central South University, Changsha, Hunan, People’s Republic of China; 2 Emergency Medicine and Difficult Diseases Institute, Second Xiangya Hospital, Central South University, Changsha, Hunan, People’s Republic of China; 3 Department of Urology, Second Xiangya Hospital, Central South University, Changsha, Hunan, People’s Republic of China; 4 Department of Ophthalmology, Second Xiangya Hospital, Central South University, Changsha, Hunan, People’s Republic of China; 5 Department of Nephrology, Second Xiangya Hospital, Central South University, Changsha, Hunan, People’s Republic of China; 6 Department of Chest Surgery, Second Xiangya Hospital, Central South University, Changsha, Hunan, People’s Republic of China; 7 Department of Endocrinology, Shenzhen People’s Hospital, The Second Clinical Medical College of Jinan University, The First Affiliated Hospital of Southern University of Science and Technology, Shenzhen, People’s Republic of China; 8 Department of Cellular Biology and Anatomy, Medical College of Georgia at Georgia Regents University and Charlie Norwood VA Medical Center, Augusta, GA 30904, USA

**Keywords:** UUO, circRNA_30032, miR-96-5p, HBEGF, KRAS

## Abstract

In this study, we investigated the role of circular RNA_30032 (circRNA_30032) in renal fibrosis and the underlying mechanisms. The study was carried out using TGF-β1-induced BUMPT cells and unilateral ureteral obstruction (UUO)-induced mice, respectively, as *in vitro* and *in vivo* models. CircRNA_30032 expression was significantly increased by 9.15- and 16.6-fold on days 3 and 7, respectively, in the renal tissues of UUO model mice. In TGF-β1-treated BUMPT cells, circRNA_30032 expression was induced by activation of the p38 mitogen-activated protein kinase signaling pathway. Quantitative real-time PCR, western blotting and dual luciferase reporter assays showed that circRNA_30032 mediated TGF-β1-induced and UUO-induced renal fibrosis by sponging miR-96-5p and increasing the expression of profibrotic proteins, including HBEGF, KRAS, collagen I, collagen III and fibronectin. CircRNA_30032 silencing significantly reduced renal fibrosis in UUO model mice by increasing miR-96-5p levels and decreasing levels of HBEGF and KRAS. These results demonstrate that circRNA_30032 promotes renal fibrosis via the miR-96-5p/HBEGF/KRAS axis and suggest that circRNA_30032 is a potential therapeutic target for treatment of renal fibrosis.

## INTRODUCTION

Chronic kidney disease (CKD) is a global health problem with an incidence rate of about 13%–15% in developed countries [1, 2]. The pathogenesis of CKD involves accumulation of extracellular matrix (ECM) proteins in the tubulointerstitium of the kidneys. [3] In the past twenty years, several promising therapeutic strategies have been tested to alleviate CKD [3–9], but the therapeutic responses have been ineffective or only partial [10]. Therefore, there is an urgent need to unravel the pathophysiological mechanisms of CKD progression in order to develop effective therapeutic strategies.

Non-coding RNAs play an important role in development of diseases by regulating gene expression, which mainly include micro RNAs (miRNAs), long noncoding RNAs(lncRNAs) and circular RNAs(circRNAs) [11]. Circular RNAs (circRNAs) are a class of non-coding RNAs that are characterized by a covalently closed loop structures without terminal 5’ and 3’ends [12, 13]. The expression patterns of specific circRNAs vary in different tissues and cell types [14, 15]. In general, circRNAs regulate gene expression by sponging their target microRNAs (miRNAs) [16]. CircRNAs play a key role in several cancers and diseases of the nervous system, cardiovascular system, lungs, liver, and kidney [17–23]. For example, knockdown of CircPDZD8 inhibited the progression of gastric cancer cells by sponging miR-197-5p and down-regulating CHD9 [24]. Hsa_circ_0002286/hsa-mir-222-5p/TRIM2 axis played a critical role in the metastasis and progression of clear cell renal cell carcinoma [25].

A recent study suggested that circACTR2 mediated high glucose-induced renal fibrosis [26]. However, the role and regulatory mechanisms of circRNAs in CKD remains largely unknown. Therefore, in this study, we investigated the role of circRNA_30032 in renal fibrosis using TGF-β1-induced BUMPT cells and unilateral ureteral obstruction (UUO)-induced mice as *in vitro* and *in vivo* models, respectively.

## RESULTS

### CircRNA_30032 expression is significantly upregulated in UUO-induced murine kidneys and TGF-β1-induced BUMPT cells

**Figure 1:**
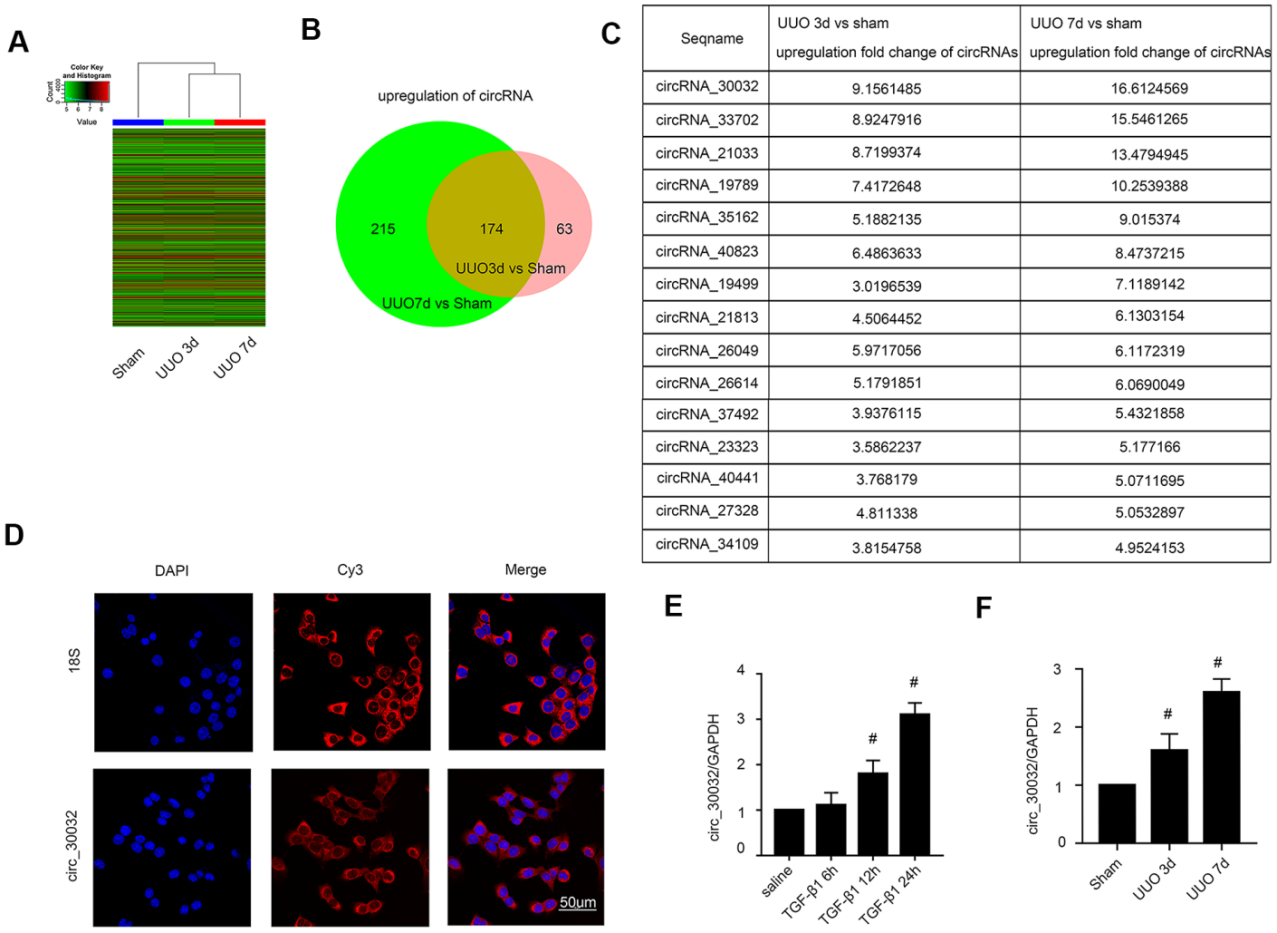
**CircRNA_30032 expression is upregulated in kidney tissues of UUO-induced C57BL/6 mice and TGF-β1 treated BUMPT cells.** (**A**) The heat map shows the expression of circRNAs in the kidney tissues of sham and UUO group mice on days 3 and 7. (**B**) The co-upregulation amount of circRNAs at (more than 2 fold changes) days 3 and 7 in UUO group vs. Sham group. (**C**) The sequence name and fold change values of significantly upregulated circRNAs (> 3 fold) in the kidney tissues of UUO group mice on days 3 and 7. (**D**) RNA-FISH images show intracellular localization of circRNA_30032 in BUMPT cells treated with saline or 5 ng/ml TGF-β1 for 24 h. (**E**, **F**) RT-qPCR analysis shows expression levels of circRNA_30032 in BUMPT cells treated with saline or 5ng/ml TGF-β1 for 6, 12 and 24 h and kidney tissues of sham and UUO group mice on days 3 and 7. Note: The data are expressed as means ± SD (n = 6); *#* denotes *p* < 0.05 for TGF-β1 groups at 12 and 24 h vs. saline group and UUO groups at days 3 and 7 vs. sham group.

We performed circRNA microarray analysis to identify significantly upregulated circRNAs in the UUO-induced renal fibrosis model mice kidney tissues at days 3 and 7 after surgery compared to those from sham controls on days 3 and 7. The expression levels of circRNAs in kidney tissue samples from sham controls and UUO model mice on days 3 and 7 were analyzed and shown in the heat map (Figure 1A). We identified 389 and 237 upregulated circRNAs on days 3 and 7, respectively, in the UUO group compared to the sham controls (Figure 1B). Among these, 174 circRNAs were upregulated by 2-fold or higher in the kidney tissues of UUO group mice at both time points (Figure 1B). Furthermore, fifteen circRNAs showed 3-fold or higher upregulation in the kidney tissues of the UUO group mice on days 3 and 7 compared to those from the sham group (Figure 1C). The most highly expressed circRNA in the kidney tissues was circRNA_30032, which showed a 9.15- and 16.6-fold higher expression in the UUO group on days 3 and 7, respectively, compared to the sham group (Figure 1C). RNA FISH (Fluorescence in site hybridization) analysis demonstrated that circRNA_30032 was localized in the cytoplasm of the BUMPT cells (Figure 1D). QRT-PCR analysis demonstrated that circRNA_30032 was upregulated in TGF-β1-treated BUMPT cells at 6, 12 and 24h, and in the kidney tissues of the UUO group mice on days 3 and 7, respectively, compared with their corresponding controls (Figure 1E, 1F). These data suggested that circRNA_30032 upregulation potentially regulates progression of renal fibrosis.

### CircRNA_30032 overexpression is promoted by activation of p38MAPK signaling pathway in TGF-β1-treated BUMPT cells

**Figure 2:**
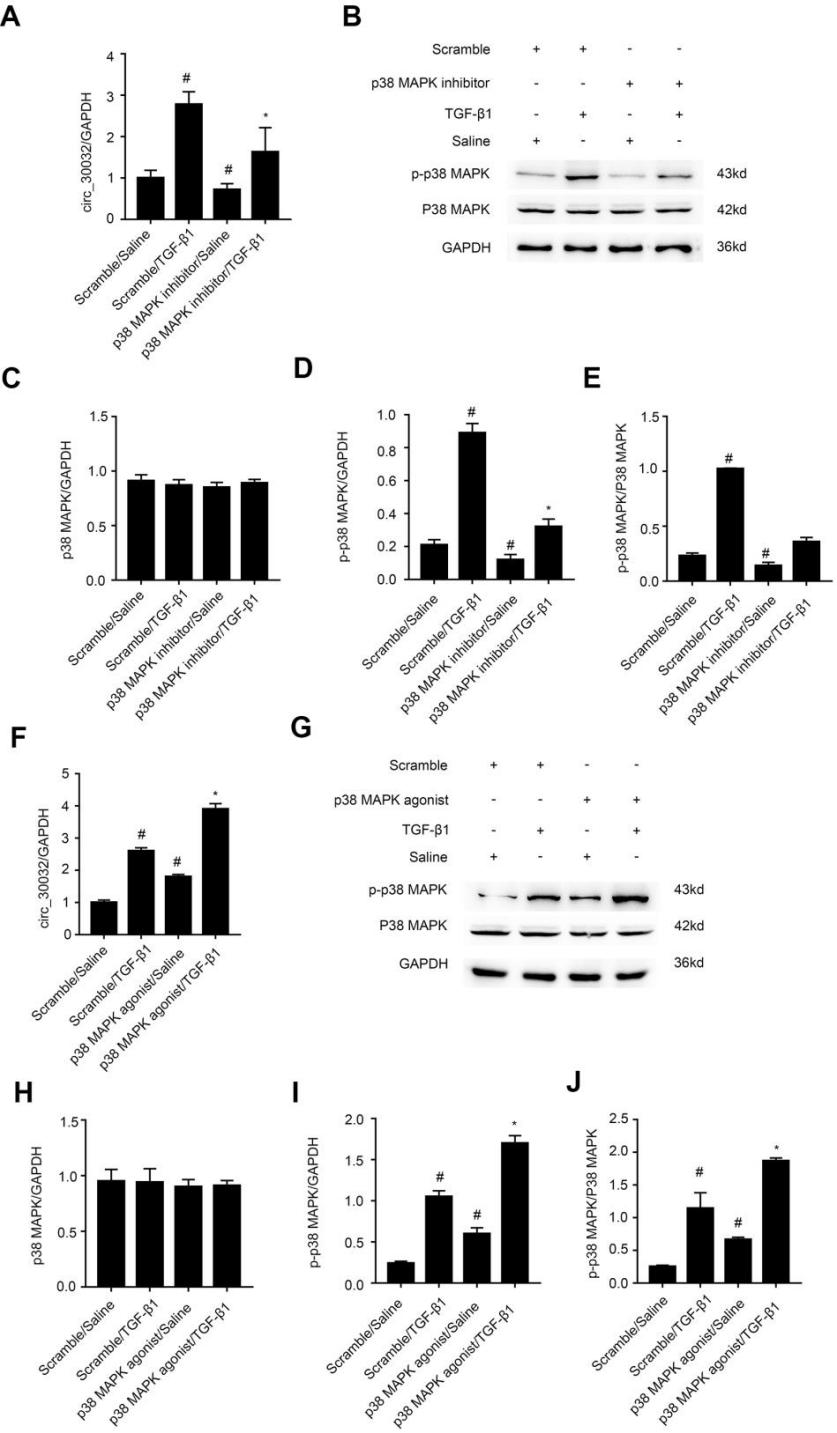
**TGF-β1 induces CircRNA_30032 in a p38MAPK-dependent manner in BUMPT cells.** (**A**, **F**) RT-qPCR analysis shows relative levels of circRNA_30032 expression in BUMPT cells treated with 5ng/mL TGF-β1 with or without p38MAPK inhibitor or agonist. (**B**, **G**) Western blot images and (**C**–**E** and **H**–**J**) quantitative analysis of p-p38MAPK, p38MAPK and GAPDH (loading control) protein levels in BUMPT cells treated with 5ng/mL TGF-β1 with or without p38MAPK inhibitor/ agonist. Note: The data are expressed as means ± SD (n = 6). *#* denotes *p* < 0.05 while comparing TGF-β1 and TGF-β1/p38MAPK inhibitor or agonist treatment groups vs. scramble group; * denotes *p* < 0.05 when comparing p38MAPK inhibitor or agonist /TGF-β1 group vs. TGF-β1 group.

We investigated the signaling pathways that regulate circRNA_30032 overexpression in TGF-β1-treated BMUPT cells by analyzing Smad, MAPKs, and p53 activation status using specific inhibitors (data not shown). The results showed that TGF-β1-induced expression of circRNA_30032 in BUMPT cells was only suppressed by SB203580, a pharmacological inhibitor of p38MAPK (Figure 2A–2E). Furthermore, activation of p38 MAPK increased the expression of circRNA_30032 (Figure 2F–2J). This suggested that activation of p38MAPK signaling pathway regulated overexpression of circRNA_30032 in TGF-β1-treated BUMPT cells.

### CircRNA_30032 silencing attenuates TGF-β1-induced expression of pro-fibrotic proteins including collagen I, collagen III, and fibronectin

**Figure 3:**
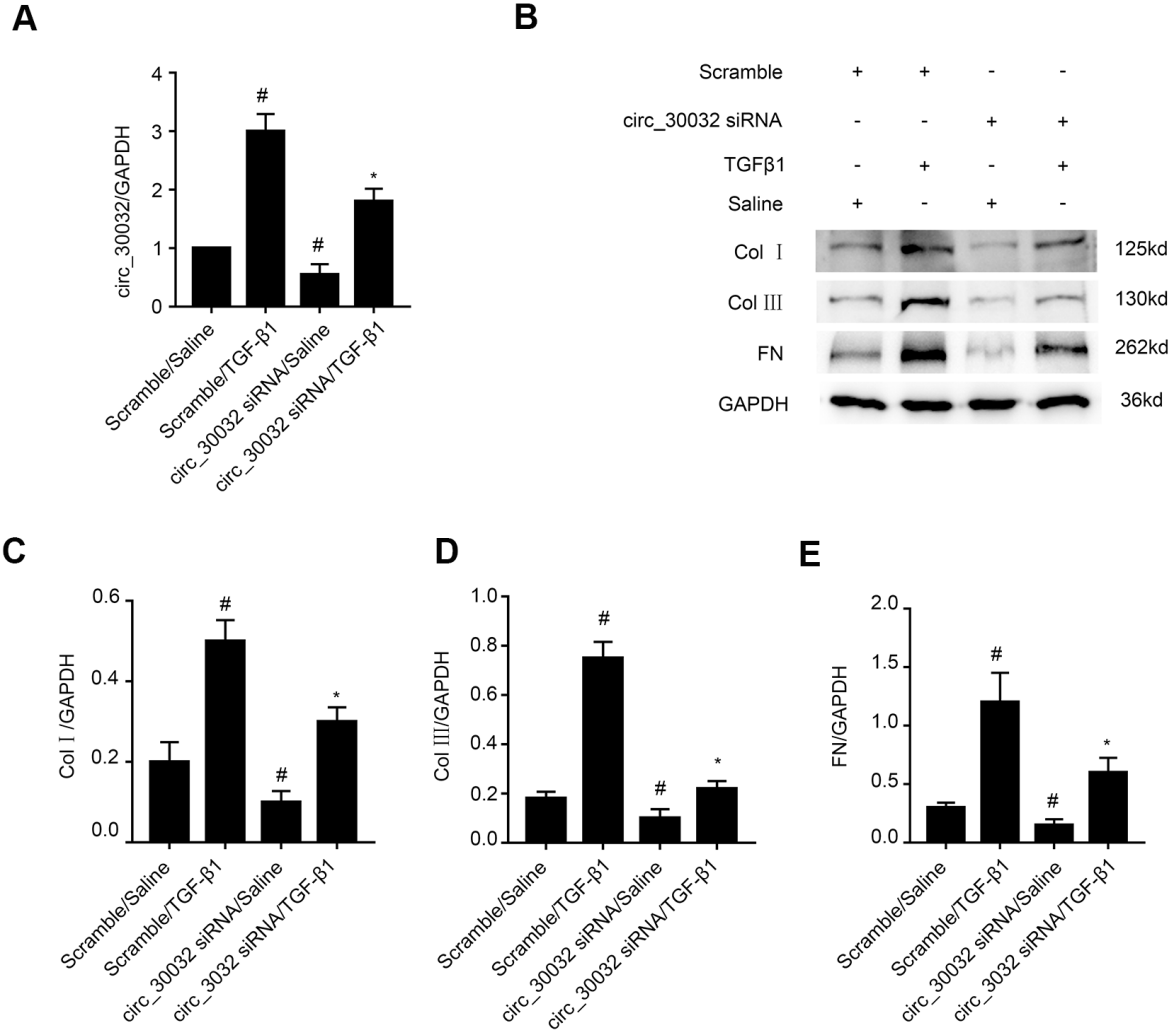
**CircRNA_30032 silencing attenuates TGF-β1-induced expression of collagen I, collagen III and fibronectin in BUMPT cells.** (**A**) RT-qPCR analysis shows the circRNA_30032 expression levels in BUMPT cells transfected with 50 nM circRNA_30032 siRNA or scrambled siRNA, and then treated with or without 5ng/mL TGF-β1 for 24 h. (**B**) Representative western blot images and (**C**–**E**) densitometric estimation of (**B**, **C**) collagen I, (**B**, **D**) collagen III, and (**B**, **E**) fibronectin protein levels in control and circRNA_30032-silenced BUMPT cells treated with or without 5ng/mL TGF-β1 for 24 h. Note: The data are expressed as means ± SD (n = 6). # denotes *p<0.05* while comparing scrambled siRNA plus TGF-β1 or circRNA_30032 siRNA plus saline groups vs. scrambled siRNA plus saline group; * denotes *p<0.05* while comparing circRNA_30032 siRNA plus TGF-β1group vs. scrambled siRNA plus TGF-β1 group.

We analyzed the role of circRNA_30032 in renal fibrosis by transfecting BUMPT cells with circRNA_30032 siRNA and studying its effects by TGF-β1 treatment. RT-qPCR analysis demonstrated that circRNA_30032-specific siRNA significantly reduced circRNA_30032 levels in control and TGF-β1-induced BUMPT cells compared to the corresponding controls (Figure 3A). Furthermore, immunoblot results demonstrated that collagen I, collagen III, and fibronectin protein levels were significantly reduced in the circRNA_30032 knockdown BUMPT cells treated with or without TGF-β1 compared to their corresponding controls (Figure 3B–3E). These results suggested that knockdown of circRNA_30032 suppressed the expression of fibrosis-related proteins in the TGF-β1-treated BUMPT cells.

### CircRNA_30032 overexpression enhances TGF-β1-induced expression of pro-fibrotic proteins including collagen I, collagen III, and fibronectin

**Figure 4:**
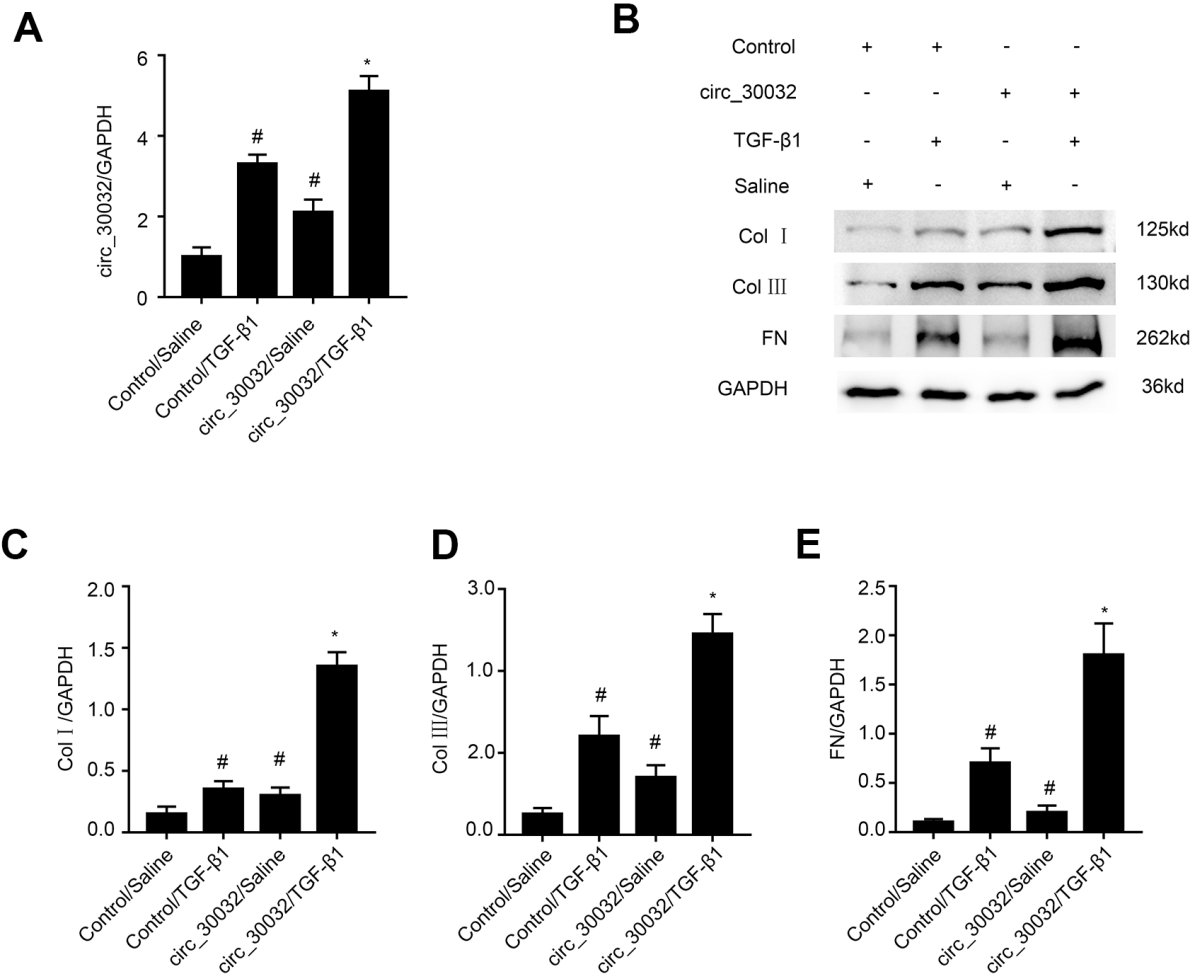
**Overexpression of circRNA_30032 enhances TGF-β1-induced expression of collagen I, collagen III, and fibronectin in BUMPT cells.** (**A**) RT-qPCR analysis shows circRNA_30032 levels in control and circRNA_30032-overexpressing BUMPT cells treated with or without TGF-β1for 24 h. (**B**) Representative western blot images and (**C**–**E**) densitometric measurements show the levels of (**B**, **C**) collagen I, (**B**, **D**) collagen III, and (**B**, **E**) fibronectin proteins in control and circRNA_30032-overxpressing BUMPT cells treated with or without TGF-β1for 24 h. Note: The data are expressed as means ± SD (n = 6). # denotes *p < 0.05* when comparing control vector plus TGF-β1 group or circRNA_30032 overexpression vector plus saline group vs. control vector plus saline group; * denotes *p < 0.05* when comparing circRNA_30032 overexpression vector plus TGF-β1 group vs. control vector plus TGF-β1 group.

RT-qPCR analysis demonstrated that circRNA_30032 overexpression enhanced circRNA_30032 expression in control and TGF-β1-treated BUMPT cells (Figure 4A). Furthermore, the levels of collagen I, collagen III, and fibronectin were significantly increased in circRNA_30032-overxpressing BUMPT cells treated with or without TGF-β1 (Figure 4B–4E). These results further demonstrated that circRNA_30032 overexpression promoted fibrosis in the TGF-β1-treated BUMPT cells.

### CircRNA_30032 directly binds to miR-96-5p in BUMPT and kidney tubular cells

**Figure 5:**
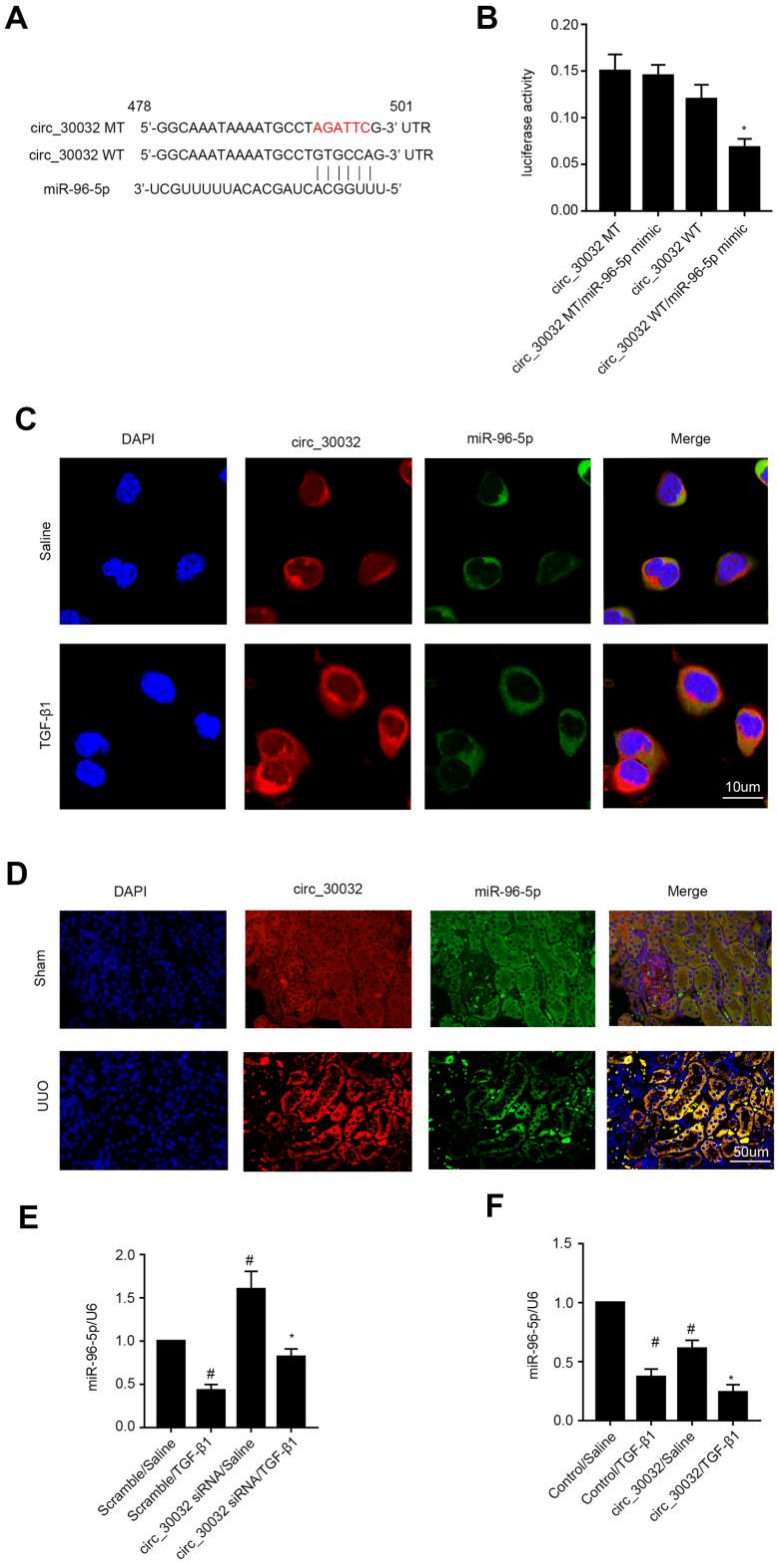
**CircRNA_30032 directly binds to miR-96-5p in BUMPT cells and murine kidney tissues.** (**A**) RegRNA 2.0 software analysis shows putative miR-96-5p binding site in the circRNA_30032 sequence. (**B**) Dual luciferase reporter assay results show relative luciferase activities in BUMPT cells co-transfected with circRNA_30032-WT (wild-type) or circRNA_30032-MT (mutant) plus miR-96-5p or scrambled miRNA. (**C**, **D**) RNA FISH images show co-localization of circRNA_30032 and miR-96-5p in kidney tissue sections from UUO-induced C57BL/6 mice and TGF-β1-treated BUMPT cells. (**E**, **F**) RT-qPCR analysis shows miR-96-5p expression levels in control, circRNA_30032-silenced, and circRNA_30032-overexpressing BUMPT cells treated with or without TGF-β1 for 24 h. Note: *#* denotes *p<0.05* when comparing scrambled miRNA plus TGF-β1 or circRNA_30032 plus saline groups vs. scrambled miRNA plus saline group; * denotes *p<0.05* when comparing circRNA_30032 WT plus miR-96-5p mimic vs. other groups, circRNA_30032 siRNA plus TGF-β1 group vs. scrambled miRNA plus TGF-β1 group.

We then performed RegRNA 2.0 software analysis and identified miR-96-5p as a potential downstream target miRNA of circRNA_30032 (Figure 5A). Dual luciferase reporter assay results demonstrated that relative luciferase activity was significantly higher in BUMPT cells co-transfected with miR-96-5p mimic and luciferase reporter vector containing wild-type circRNA_30032 (circRNA_30032-WT) and significantly suppressed in BUMPT cells transfected with miR-96-5p mimic and luciferase reporter vector containing mutant circRNA_30032 (circRNA_30032-MT) (Figure 5B). Co-localization experiments showed that circRNA_30032 interacted with miR-96-5p in the cytoplasm of BUMPT cells treated with or without TGF-β1 as well as in the renal tubular cells of the kidneys from the sham and UUO group mice (Figure 5C, 5D). Furthermore, circRNA_30032 silencing increased the levels of miR-96-5p in the TGF-β1-induced BUMPT cells, whereas, circRNA_30032 overexpression significantly suppressed the expression of miR-96-5p in the TGF-β1-induced BUMPT cells (Figure 5E, 5F). These data confirmed that miR-96-5p was a direct target of circRNA_30032.

### MiR-96-5p mimics suppress TGF-β1-induced expression of collagen I, collagen III, and fibronectin in BUMPT cells

**Figure 6:**
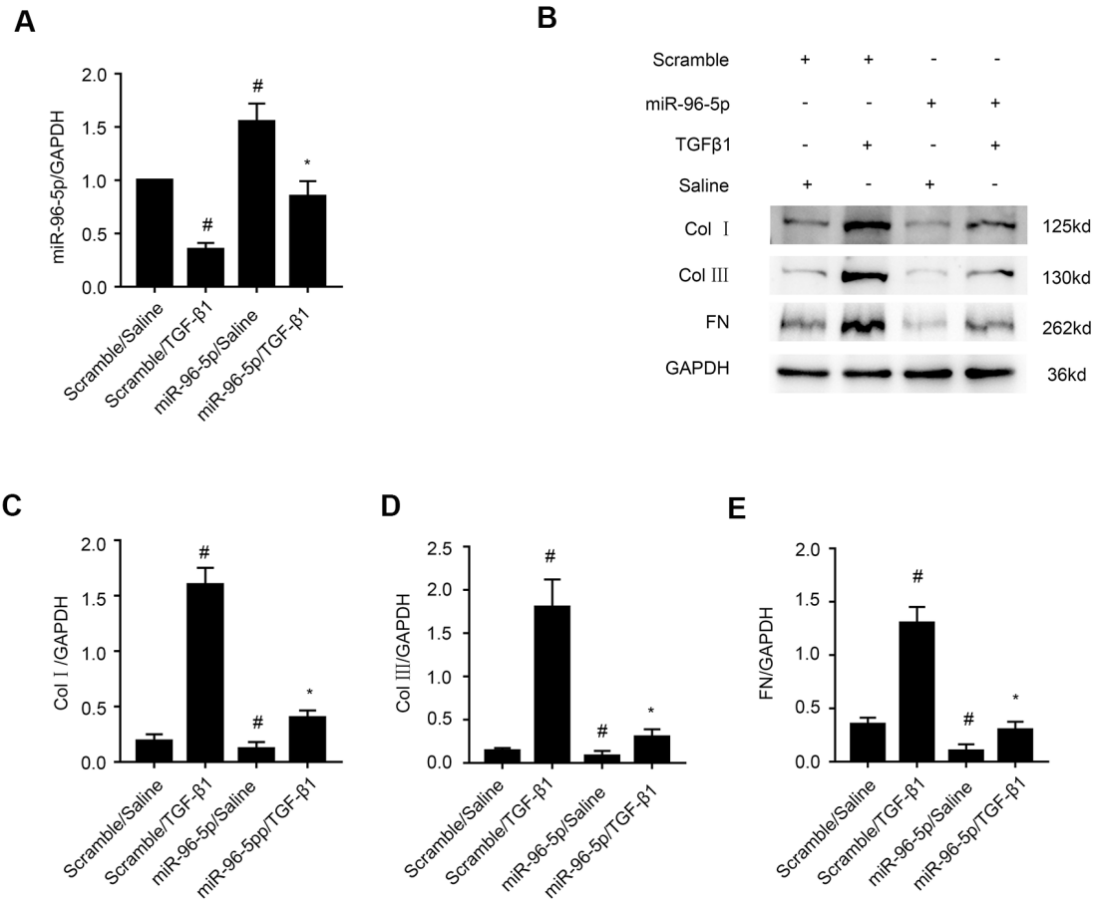
**MiR-96-5p inhibits TGF-β1-induced expression of collagen I, collagen III, and fibronectin.** (**A**) RT-qPCR analysis shows miR-96-5p expression levels in BUMPT cells transfected with 100 nM miR-96-5p mimics or scrambled miRNA, and then treated with or without TGF-β1 for 24h. (**B**) Representative western blot images and (**C**–**E**) densitometric measurements show (**B**, **C**) collagen I, (**B**, **D**) collagen III, and (**B**, **E**) fibronectin protein levels in BUMPT cells transfected with 100 nM miR-96-5p mimics or scrambled miRNA, and then treated with or without TGF-β1 for 24h. Note: The data are expressed as means ±SD (n = 6). # denotes *p < 0.05* when comparing scrambled miRNA plus TGF-β1 group or miR-96-5p mimics plus saline group vs. scrambled miRNA plus saline group; * denotes *p<0.05* when comparing miR-96-5p mimics plus TGF-β1group vs. scrambled miRNA plus TGF-β1 group.

A recent study reported that miR-96-5p played an anti-fibrosis role in diabetic kidney disease (DKD) [27]. In our study, RT-qPCR analysis showed that miR-96-5p levels were significantly increased in miR-96-5p mimic-transfected BUMPT cells that were treated with or without TGF-β1 (Figure 6A). Furthermore, miR-96-5p mimic suppressed the levels of collagen I, collagen III, and fibronectin in BUMPT cells treated with or without TGF-β1 (Figure 6B–6E). These results confirmed that miR-96-5p inhibited expression of fibrosis-related proteins in TGF-β1 treated BUMPT cells.

### HBEGF and KRAS transcripts are direct downstream targets of miR-96-5p

**Figure 7:**
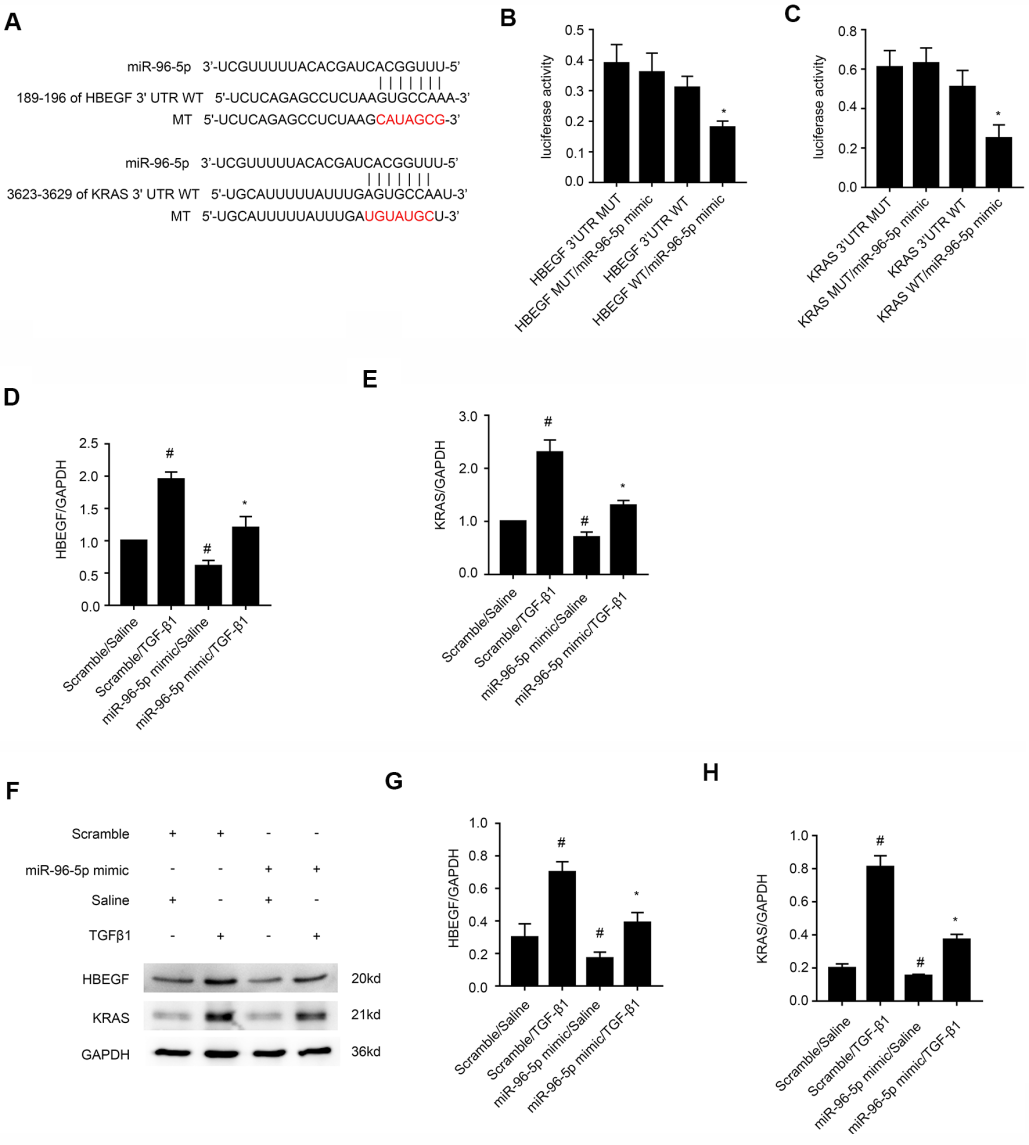
**MiRNA-96-5p directly binds to the 3’UTR of HBEGF and KRAS mRNAs in BUMPT cells.** (**A**) TargetScan database analysis shows putative miR-96-5p binding sites in the 3’UTR of HBEGF and KRAS mRNAs. (**B**, **C**) Dual luciferase reporter assay results show relative luciferase activities in BUMPT cells co-transfected with luciferase reporter vectors carrying wild-type (WT) or mutant (MUT) 3’ UTR’s of (**B**) HBEGF and (**C**) KRAS (**C**) plus miR-96-5p or miR-NC. (**D**, **E**) RT-qPCR analysis shows (**D**) HBEGF and (**E**) KRAS mRNA levels in BUMPT cells transfected with 100 nM miR-96-5p mimics or miR-NC, and then treated with TGF-β1 for 24h. (**F**) Representative western blot images and (**G**, **H**) densitometric measurements show the levels of (**G**) HBEGF and (**H**) KRAS proteins in BUMPT cells transfected with 100 nM miR-96-5p mimics or miR-NC, and then treated with TGF-β1 for 24h. Note: The data are expressed as means ± SD (n = 6). # denotes *p < 0.05* when comparing miR-NC plus TGF-β1or miR-96-5p mimic with saline groups vs. miR-NC plus saline group; * denotes *p < 0.05* when comparing miR-96-5p mimics plus TGF-β1group vs. miR-NC plus TGF-β1 group and KRAS-WT or HBEGF-WT plus miR-96-5p mimic vs. other groups.

A study by Wang et al., demonstrated the anti-fibrosis role of miR-96-5p [27], but the regulatory mechanisms underlying these effects are not clear. Therefore, we analyzed the TargetScan database and identified HBEGF and KRAS as potential downstream target genes of miR-96-5p (Figure 7A). Dual luciferase reporter assay showed that the relative luciferase activity was significantly higher in BMPT cells co-transfected with miR-96-5p mimic and luciferase reporter vector containing wild-type HBEGF (HBEGF-WT) or KRAS (KRAS-WT) 3’UTRs, but was significantly reduced in BUMPT cells co-transfected with miR-96-5p mimic and luciferase reporter vectors with mutant HBEGF (HBEGF-MUT) or KRAS (KRAS-MUT) 3’UTRs (Figure 7B, 7C). Finally, miR-96-5p mimic transfection significantly reduced both mRNA and proteins levels of HBEGF and KRAS in BUMPT cells treated with or without TGF-β1 (Figure 7D–7H). These data demonstrated that HBEGF and KRAS were direct targets of miR-96-5p.

### MiR-96-5p mediates pro-fibrotic effects of circRNA_30032

**Figure 8:**
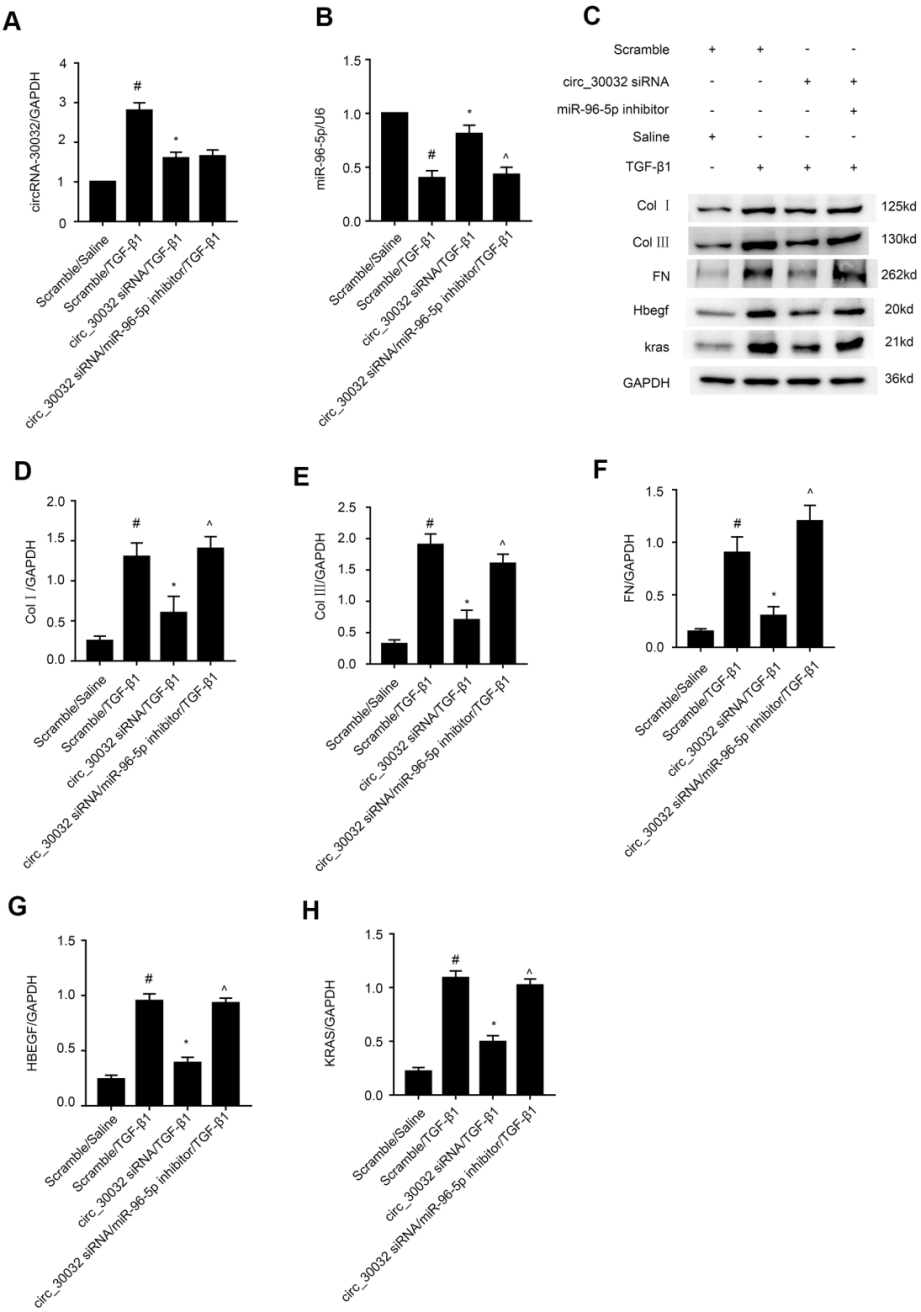
**CircRNA_30032 promotes expression of pro-fibrosis factors in TGF-β1 treated BUMPT cells by suppressing miR-96-5p.** (**A**, **B**) RT-qPCR analysis shows (**A**) circRNA_30032 and (**B**) RT-qPCR analysis of miR-96-5p expression. (**C**) Representative western blot images and (**D**, **H**) densitometric measurements show levels of (**C**, **D**) collagen I, (**C**, **E**) collagen III, (**C**, **F**) fibronectin, (**C**, **G**) HBEGF, and (**C**, **H**) KRAS proteins in BUMPT cells co-transfected with 100 nM circRNA_30032 siRNA plus anti-miR-96-5p or anti-miRNA, and then treated with TGF-β1 for 24h. Note: The data are expressed as means ± SD (n = 6). # denotes *p < 0.05* when comparing anti-miRNA plus TGF-β1 group vs. anti-miRNA plus saline group; * denotes *p < 0.05* when comparing circRNA_30032 siRNA plus TGF-β1 group vs. anti-miRNA plus TGF-β1 group; ^ denotes *p < 0.05* when comparing circRNA_30032 siRNA plus anti-miR-96-5p plus TGF-β1 group vs. circRNA_30032 siRNA plus TGF-β1 group.

We further investigated if miR-96-5p mediated the pro-fibrotic effects of circRNA_30032 in TGF-β1-treated BUMPT cells. RT-qPCR analysis confirmed that circRNA_30032 levels were significantly reduced in circRNA_30032 silenced BUMPT cells with or without TGF-β1 treatment, whereas, miR-96-5p levels were significantly reduced in miR-96-5p inhibitor-transfected BUMPT cells with or without TGF-β1 treatment (Figure 8A, 8B). Western blotting results demonstrated that circRNA_30032 silencing attenuated the expression of collagen I, collagen III, fibronectin, HBEGF and KRAS in TGF-β1-treated BUMPT cells, but these effects were reversed in the miR-96-5p inhibitor-transfected BUMPT cells treated with TGF-β1 (Figure 8C–8H). These data confirmed that circRNA_30032 promoted renal fibrosis by sponging miR-96-5p.

### Inhibition of circRNA_30032 attenuates UUO-induced the renal fibrosis

**Figure 9:**
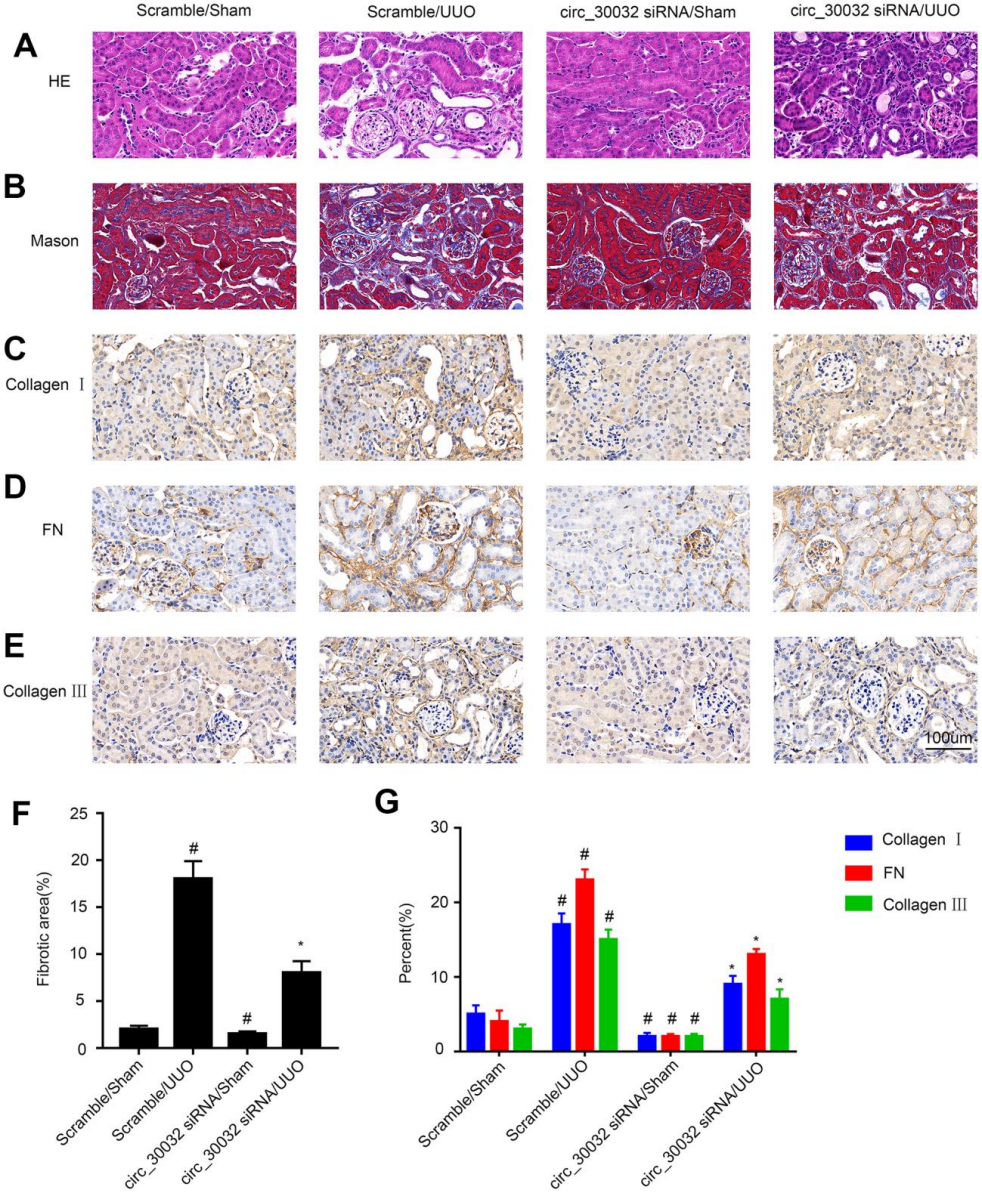
**CircRNA_30032 silencing attenuates UUO-induced renal fibrosis in male C57BL/6 mice.** (**A**) H&E and (**B**) Masson staining images of kidney tissue sections. (**C**–**E**) Representative immunohistochemical (IHC) staining images show expression patterns of (**C**) collagen I, (**D**) collagen III, and (**E**) fibronectin in the kidney tissue sections of control and circRNA-30032 silenced UUO and sham group mice. (**F**) Quantification of fibrotic area (%) in the kidney cortex of control and circRNA-30032 silenced UUO and sham group mice based on Masson staining. (**G**) Quantitative analysis of IHC data shows collagen I, collagen III, and fibronectin levels in the kidney tissue sections of control and circRNA-30032 silenced UUO and sham group mice. Note: magnification, ×200; scale bar = 50μm. The data are expressed as means ± SD (n=6). # denotes *p < 0.05* when comparing circRNA_30032 siRNA plus sham group or scrambled siRNA plus UUO group vs. scrambled siRNA plus sham group; * denotes *p<0.05* when comparing circRNA_30032 siRNA plus UUO group vs. scrambled siRNA plus UUO group.

Next, we analyzed if knockdown of circRNA_30032 attenuated renal fibrosis in UUO-induced renal fibrosis model mice. H&E staining analysis showed that knockdown of circRNA_30032 decreased UUO-induced tubular dilation and atrophy (Figure 9A). Masson’s trichrome staining demonstrated that knockdown of circRNA_30032 decreased UUO-induced interstitial expansion and collagen deposition in the kidneys (Figure 9B, 9F). Immunohistochemical staining analysis demonstrated that knockdown of circRNA_30032 reduced the expression and deposition of collagen I, collagen III, and fibronectin in the kidneys of UUO-induced renal fibrosis model mice compared to the corresponding controls (Figure 9C–9E, 9G). These data suggested that circRNA_30032 mediated progression of UUO-induced renal fibrosis.

### CircRNA_30032 silencing inhibits UUO-induced accumulation of ECM proteins in the kidney via miR-96-5p/ HBEGF/KRAS axis

**Figure 10:**
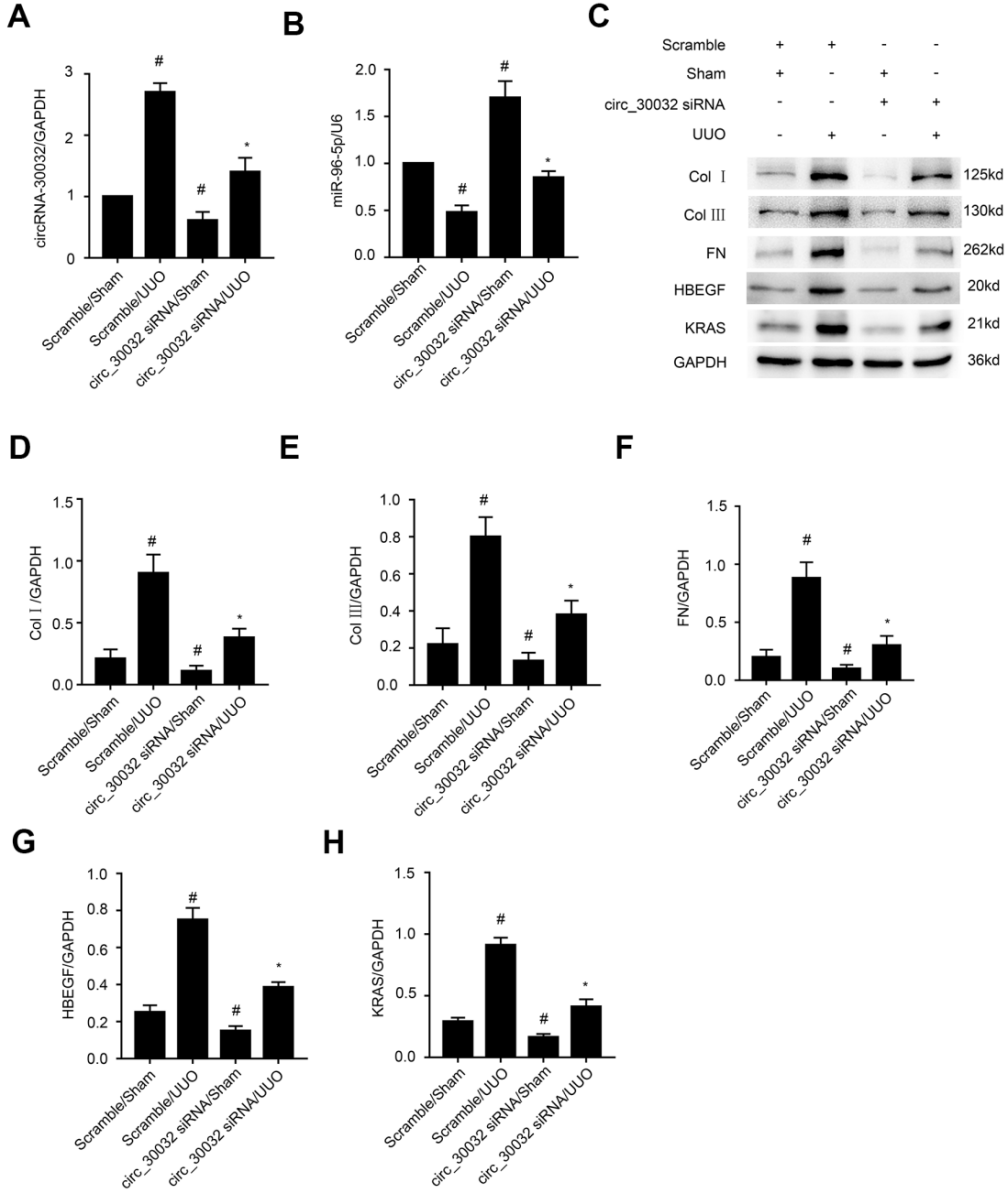
**CircRNA_30032 siRNA ameliorates UUO-induced expression of collagen I, collagen III and fibronectin by targeting miR-96-5p/HBEGF/KRAS axis.** (**A**, **B**) RT-qPCR analysis shows (**A**) circRNA_30032 and (**B**) miR-96-5p levels in the kidney tissues harvested from UUO and sham group mice injected with scrambled siRNA or circRNA_30032 siRNA. (**C**) Representative western blot images and (**D**–**H**) densitometric measurements show the levels of (**C**, **D**) collagen I, (**C**, **E**) collagen III, (**C**, **F**) fibronectin, (**C**, **G**) HBEGF, and (**C**, **H**) KRAS proteins in the kidney tissues harvested from UUO and sham group mice injected with scrambled siRNA or circRNA_30032 siRNA. GAPDH was used as a loading control. Note: The data are expressed as means ± SD (n =6). # denotes *p < 0.05* when comparing circRNA_30032 siRNA plus sham group or scrambled siRNA plus UUO group vs. scrambled siRNA plus sham group; * denotes *p<0.05* when comparing circRNA_30032 siRNA plus UUO group vs. scrambled siRNA plus UUO group.

We then investigated the molecular mechanisms underlying the pro-fibrotic effects of circRNA_30032 in the UUO-induced renal fibrosis model mice. RT-qPCR analysis showed that interveinal injection of circRNA_30032 siRNA significantly reduced the levels of circRNA_30032 and increased the levels of miR-96-5p in the kidney tissues of UUO group mice compared to the sham controls (Figure 10A, 10B). Moreover, immunoblot analysis demonstrated that circRNA_30032 silencing reduced the levels of collagen I, collagen III, fibronectin, HBEGF, and KRAS proteins in the kidney tissues of UUO group mice (Figure 10C–10H). These data demonstrated that circRNA_30032 promoted UUO-induced renal fibrosis via miR-96-5p/ HBEGF/KRAS axis.

## DISCUSSION

**Figure 11:**
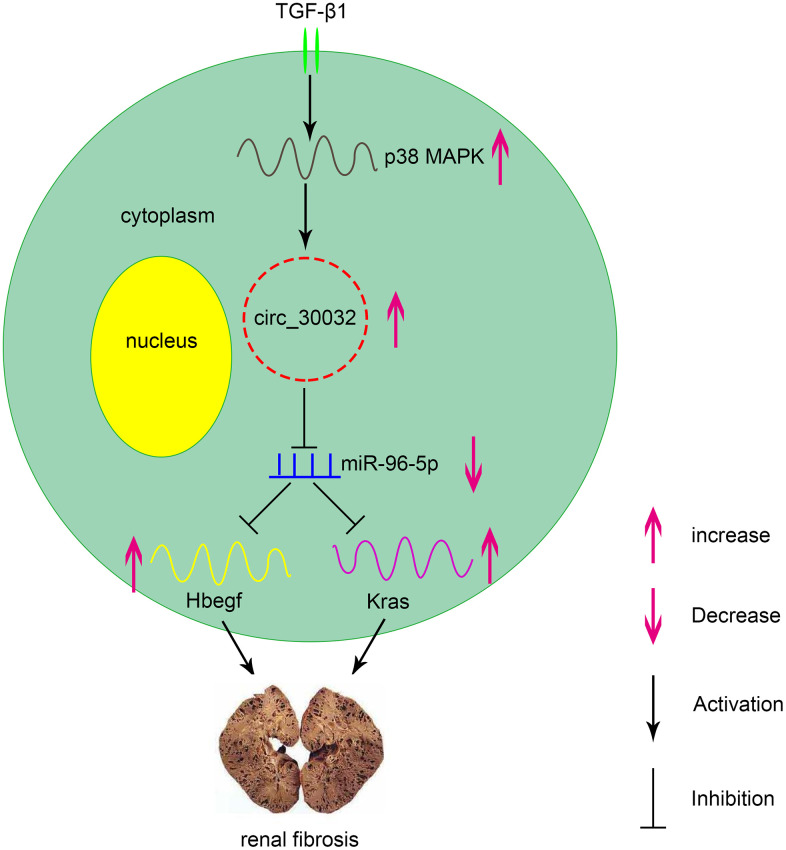
**Molecular mechanism underlying the role of circRNA_30032 in TGF-β1-induced renal fibrosis.** TGF-β1 induces expression of circRNA_30032 via p38 MAPK signaling pathway. CircRNA_30032 promotes renal fibrosis by sponging miR-96-5p and subsequently increasing the expression of HBEGF and KRAS.

CircRNAs have been recognized as vital regulators in multiple diseases. In the current study, we demonstrated that circRNA_30032 mediated TGF-β1-induced renal fibrosis in BUMPT cells and in kidney tissues of UUO-induced mice by sponging miR-96-5p and inducing the expression of HBEGF and KRAS (Figure 11). Moreover, knockdown of circRNA_30032 attenuated UUO-induced renal fibrosis by regulating the miR-96-5p/ HBEGF/KRAS axis.

Recently, evidence suggesting circRNAs have an impact on renal diseases increased. Liu et al., demonstrated that circ_0080425 suppressed renal fibrosis in diabetic nephropathy by regulating miR-24-3p/FGF11 axis [28]. Hu et al., found that circRNA_15698 aggravates high glucose-induced ECM accumulation in mesangial cells via miR-185/TGF-β1 axis [29]. However, the roles of other circRNAs in renal fibrosis have not been investigated. We identified 174 upregulated circRNAs in the kidney tissues of the UUO group mice on days 3 and 7. Among these, circRNA_30032 showed the highest expression (Figure 1A–1C and Supplementary Table 1). CircRNA_30032 was mainly expressed in the cytoplasm of the BUMPT cells (Figure 1D) and regulated by the p38MAPK signaling pathway (Figure 2). CircRNA_30032 silencing attenuated TGF-β1-induced fibrosis in BUMPT cells, but these effects were reversed by overexpressing circRNA_30032 (Figures 3, 4). Moreover, silencing of circRNA_30032 attenuated UUO-induced renal fibrosis in mice (Figure 9). Overall, our data demonstrated that circRNA_30032 promoted renal fibrosis via miR-96-5p/ HBEGF/KRAS axis (Figure 11).

CircRNAs are competitive endogenous RNAs (ceRNAs) that regulate gene expression by sponging their target miRNAs [30–32]. For example, circular RNA YAP1 protects against ischemic injury by regulating the miRNA-21-5p/PI3K/AKT/mTOR axis [33]. CircLRP6 was found to act as a sponge for miR-205 to regulate proliferation, oxidative stress, ECM accumulation, and inflammation in mesangial cells [34]. In present study, we demonstrated that miR-96-5p was a direct target of circRNA_30032 (Figure 5B). Moreover, co-localization experiments demonstrated that circRNA_30032 interacted with miR-96-5p in the cytoplasm of BUMPT cells and the murine kidney tubular cells (Figure 5C, 5D). Our results demonstrated that circRNA_30032 acted as a ceRNA by sponging miR-96-5p (Figure 5E, 5F).

Our study demonstrated that miR-96-5p regulated the expression levels of pro-fibrotic proteins, namely, collagen I, collagen III, and fibronectin in the TGF-β1-induced BUMPT cells (Figure 6). This further supported previous findings by Yang et al., who reported that progression of renal fibrosis was regulated by miR-96-5p in DKD [35]. Furthermore, we demonstrated that HBEGF and KRAS mRNAs were direct targets of miR-96-5p using the dual luciferase reporter assay (Figure 7A–7C). These findings were supported by RT-qPCR and immunoblot experiments, which showed that miR-96-5p significantly suppressed the expression of HBEGF and KRAS in BUMPT cells with or without TGF-β1 treatment (Figure 7D–7H). Previous studies have demonstrated that both HBEGF and KRAS promote renal fibrosis [36–38]. We also showed that knock-down of circRNA_30032 decreased TGF-β1-induced fibrosis in the BUMPT cells by downregulating HBEGF and KRAS, but these effects were reversed by the miR-96-5p inhibitor (Figure 8). Moreover, circRNA_30032 silencing suppressed UUO-induced renal fibrosis via the miR-96-5p/HBEGF /KRAS axis (Figures 9, 10).

In conclusion, our study demonstrates that circRNA_30032 promotes TGF-β1-induced and UUO-induced renal fibrosis via miR-96-5p/HBEGF /KRAS axis and p38MAPK signaling pathway (Figure 11). These data suggest that circRNA_30032 is a potential therapeutic target for renal fibrosis disease.

## MATERIALS AND METHODS

### Antibodies and reagents

Primary antibodies against p38 MAPK (Cat. No. ab31828), collagen I (Cat. No. ab34710), collagen III (Cat. No. ab7778), fibronectin (Cat. No.ab2413), HBEGF (Cat. No. ab92620), and KRAS (Cat. no. ab180772) were purchased from Abcam (Cambridge, MA, USA). Anti- GAPDH antibody (Cat. No. 10494-1-AP) and TGF-β were obtained from Proteintech North America (Rosemont, IL, USA). Anti-phospho-p38MAPK antibody (Cat. No. 4511) was purchased from Cell Signaling Technology (Danvers, MA, USA). The luciferase assay kit was purchased from BioVision (Milpitas, CA, USA). All plasmids used in this study were generated by Vigene Biosciences (Jinan, Shangdong, China). The p38 MAPK inhibitor, SB203580 (Cat. No. HY-10256) and p38MAPK agonist (Cat. #HY-N0674A/CS-6061) were purchased from MedChemExpress USA (Deer Park, NJ, USA).

### Cell culturing, transfections, and treatments

BUMPT cells were grown in Dulbecco’s Modified Eagle’s Medium (DMEM; Thermo Fisher Scientific) containing 10% fetal bovine serum (FBS), penicillin (100U/ml) and streptomycin (100μg/ml) in a humidified incubator maintained at 37° C and 5% CO_2_. We transfected BUMPT cells with 100 nM anti-miR-96-5p, 100 nM miR-96-5p mimic, 100 nM circ30032 siRNA, or 100 nM negative control siRNA (Ruibo, Guangzhou, China) using Lipofectamine 2000 (Life Technologies, Carlsbad, CA, USA) and cultured them for 24 h. Then, the cells were incubated overnight in serum-free medium and subsequently cultured in medium with or without 5 ng/ml TGF-β1 for another 24 hours.

### Dual luciferase reporter assays

The dual luciferase reporter assay was carried out as previously described [39–42]. Briefly, the wild-type and mutant circRNA_30032, and wild-type and mutant 3’UTR’s of HBEGF and KRAS were cloned into the pmir GLO dual-luciferase target expression vector, and co-transfected with miR-96-5p mimics into the BUMPT cells. Renilla luciferase (RLuc) was used as an internal control. The luciferase reporter activities were measured after 48 h using SpectraMaxM5 (Molecular Devices, Sunnyvale, CA, USA), and normalized relative to the RLuc signal [43].

### UUO-induced renal fibrosis model mice

The UUO-induced renal fibrosis murine model was established by ligating the left ureter of male 10-12 week old C57BL/6 mice as previously described [8, 9, 44]. The mice were then intravenously injected twice a week with 15 mg/kg circRNA_30032 siRNA or saline. The animal experiments were performed according to the protocols approved by the Institutional Committee for the Care and Use of Laboratory Animals of Second Xiangya Hospital (China). All animals were given free access to food and water at all times, and housed under a 12-hour light/dark cycle.

### Histological and immunohistochemical staining

H&E staining was used to identify histological changes in the kidney tissue specimens. The degree of fibrosis was assessed by Masson’s trichrome staining. Immunohistochemical analysis was performed by staining kidney tissue sections with primary antibodies against collagen I (1:100 dilution), collagen III (1:100 dilution), and fibronectin (1:100 dilution) as previously described [45]. The stained sections were evaluated and photographed using a Olympus microscope equipped with UVepi-illumination.

### Real-Time qPCR

Total RNA from the BUMPT cells and kidney tissues of C57BL/6J mice were extracted using Trizol (Invitrogen, Carlsbad, CA, USA) according to the manufacturer’s instructions. Total RNA (40 ng) was then reverse transcribed with the M-MLV Reverse Transcriptase (Invitrogen). The expression levels of miRNA-96-5p, circRNA_30032, GAPDH, and U6 transcripts were determined by performing qPCR assay in an Opticon Real-time Cycler (MJ Research, Waltham, MA, USA) using the Bio-Rad IQ SYBR Green Supermix (Hercules, CA) according to the manufacturer’s protocol. The sequences of miR-96-5p and circRNA_30032 were retrieved from the Gen Bank database with gene IDs 723886 and 15975 (NM_010508), respectively, and are listed in the Supplementary Data file. The primers used for q-PCR analysis were as follows: circRNA_30032: 5’- CTCTTCAGGGCACAGTGGCT-3’ (forward) and 5’- TGCTGTTCCCTTCCTCTGCT-3’ (reverse); miR-96-5p:5’- GCGTTTGGCACTAGCACATT-3’ (forward) and 5’- AGTGCAGGGT CCGAGGTATT-3’ (reverse); GAPDH: 5’-GGTCTCCTCTGACTTCACA-3’ (forward) and 5’-GTGAGGGTCTCTCTCTTCCT-3’ (reverse); U6 primers were designed as described previously [39, 46]. The relative levels of miR-96-5p and circRNA_30032 were determined using the 2^-ΔΔCt^ method using U6 RNA and GAPDH mRNA as controls, respectively.

### Western blotting

Equal amounts of total protein lysates were resolved by SDS-PAGE and electrophoretically transferred onto a nitrocellulose membrane (Amersham, Buckinghamshire, UK) as previously described [47–49]. The membranes were blocked using 5% skimmed milk followed by overnight incubation at 4° C with primary antibodies against p-p38MAPK, p38MAPK, collagen I, collagen III, fibronectin, HBEGF and KRAS. Then, the blots were incubated with the corresponding HRP-conjugated secondary antibodies and detected as previously described [50]. The relative amounts of various proteins were estimated using GAPDH as the loading control.

### Fluorescence *in situ* hybridization (FISH)

The fluorescent labeled probes for detecting circRNA_30032 and miR-96-5p were synthesized by Ruibo company (Guangzhou, China). Briefly, the BUMPT cells and murine kidney sections were hybridized overnight with fluorescent labeled probes to detect circRNA_30032 (CY3) and miR-96-5p. DAPI was used to stain the nuclei [46]. The slides were photographed using a laser scanning confocal microscope.

### Statistical analysis

The differences between groups were compared using two-tailed Student’s t tests. The differences between multiple groups were compared by one-way ANOVA. All quantitative data were expressed as means ± SD and p*< 0.05* was considered statistically significant. All statistical analyses were performed with the SPSS package (SPSS) and GraphPad Prism software (GraphPad Prism Software).

## Supplementary Material

Supplementary Data

Supplementary Table 1
