# Supplementary material for: CircRNA_30032 promotes renal fibrosis in UUO model mice via miRNA-96-5p/HBEGF/KRAS axis
Source: Aging (Albany NY). 2021 May 11;13(9):12780–99. doi: 10.18632/aging.202947 (PMC8148471; doi:10.18632/aging.202947)
Supplement: Supplementary Data [file aging-13-202947-s001.pdf]

SUPPLEMENTARY DATA

The sequences of circRNA_30032

mmu_circRNA_30032

AAAAGACGAGGCGAAGTGGTTAAAAGTGCCTG
AATGTCAACATACTACAACGACCAAGTG
TGAATTCTCTTTACTGGACACAAATGTGTATAT
CAAAACACAGTTTCGTGTCAGAGCAGA
GGAAGGGAACAGCACATCTTCGTGGAATGAGG
TTGATCCGTTTATTCCATTCTACACAGC
TCACATGAGCCCCCCAGAAGTACGTTTAGAAGC
TGAAGATAAAGCCATACTAGTCCACAT
CTCtcctcccggacaagacgggaacatgtgggcactggagaaACCTT
CCTTCAGTTACAC
CATACGAATCTGGCAGAAGTCTTCCAGTGACAA
AAAAACTATTAACTCTACGTATTATGT
AGAAAAGATACCAGAACTCTTGCCAGAGACTA
CTTACTGTTTAGAAGTTAAAGCAATACA
TCCGTCACTTAAGAAACACAGCAATTACAGCAC
TGTGCAGTGTATAAGCACCACAGTGGC
AAATAAAATGCCTGTGCCAGGAAATCTCCAAG
TGGATGCCCAAGGCAAGAGCTATGTCCT
GAAATGGGACTACATTGCGTCTGCAGACGTGCT
CTTCAGGGCACAGTGGCTTCC.
