# Supplementary material for: CircRNA_30032 promotes renal fibrosis in UUO model mice via miRNA-96-5p/HBEGF/KRAS axis
Source: Aging (Albany NY). 2021 May 11;13(9):12780–99. doi: 10.18632/aging.202947 (PMC8148471; doi:10.18632/aging.202947)
Supplement: Supplementary Table 1 [file aging-13-202947-s002.docx]

**Supplementary Table 1. 174 co-upregulated circRNAs in UUO model at days 3 and 7.**

| mmu_circRNA_31043 |
| --- |
| mmu_circRNA_40001 |
| mmu_circRNA_40395 |
| mmu_circRNA_21172 |
| mmu_circRNA_25332 |
| mmu_circRNA_22903 |
| mmu_circRNA_19001 |
| mmu_circRNA_41361 |
| mmu_circRNA_41718 |
| mmu_circRNA_19789 |
| mmu_circRNA_21813 |
| mmu_circRNA_22355 |
| mmu_circRNA_31954 |
| mmu_circRNA_015487 |
| mmu_circRNA_41205 |
| mmu_circRNA_23323 |
| mmu_circRNA_22102 |
| mmu_circRNA_37492 |
| mmu_circRNA_32184 |
| mmu_circRNA_37818 |
| mmu_circRNA_37497 |
| mmu_circRNA_38378 |
| mmu_circRNA_41832 |
| mmu_circRNA_43447 |
| mmu_circRNA_19534 |
| mmu_circRNA_40796 |
| mmu_circRNA_41436 |
| mmu_circRNA_018777 |
| mmu_circRNA_19557 |
| mmu_circRNA_42341 |
| mmu_circRNA_38014 |
| mmu_circRNA_42481 |
| mmu_circRNA_41221 |
| mmu_circRNA_001254 |
| mmu_circRNA_28778 |
| mmu_circRNA_30032 |
| mmu_circRNA_23306 |
| mmu_circRNA_012978 |
| mmu_circRNA_30666 |
| mmu_circRNA_36807 |
| mmu_circRNA_25320 |
| mmu_circRNA_014445 |
| mmu_circRNA_018406 |
| mmu_circRNA_000036 |
| mmu_circRNA_005865 |
| mmu_circRNA_38102 |
| mmu_circRNA_002100 |
| mmu_circRNA_24439 |
| mmu_circRNA_30665 |
| mmu_circRNA_31759 |
| mmu_circRNA_21120 |
| mmu_circRNA_38341 |
| mmu_circRNA_26614 |
| mmu_circRNA_29487 |
| mmu_circRNA_42852 |
| mmu_circRNA_32945 |
| mmu_circRNA_25329 |
| mmu_circRNA_010484 |
| mmu_circRNA_40689 |
| mmu_circRNA_30296 |
| mmu_circRNA_35215 |
| mmu_circRNA_31436 |
| mmu_circRNA_26966 |
| mmu_circRNA_42362 |
| mmu_circRNA_21181 |
| mmu_circRNA_22217 |
| mmu_circRNA_31187 |
| mmu_circRNA_24153 |
| mmu_circRNA_29069 |
| mmu_circRNA_37684 |
| mmu_circRNA_44660 |
| mmu_circRNA_28817 |
| mmu_circRNA_002319 |
| mmu_circRNA_014597 |
| mmu_circRNA_35214 |
| mmu_circRNA_012114 |
| mmu_circRNA_35213 |
| mmu_circRNA_22256 |
| mmu_circRNA_28794 |
| mmu_circRNA_43841 |
| mmu_circRNA_30344 |
| mmu_circRNA_43625 |
| mmu_circRNA_22310 |
| mmu_circRNA_26056 |
| mmu_circRNA_19186 |
| mmu_circRNA_22793 |
| mmu_circRNA_42016 |
| mmu_circRNA_012933 |
| mmu_circRNA_29625 |
| mmu_circRNA_31762 |
| mmu_circRNA_42723 |
| mmu_circRNA_24179 |
| mmu_circRNA_19180 |
| mmu_circRNA_22030 |
| mmu_circRNA_25839 |
| mmu_circRNA_30845 |
| mmu_circRNA_43825 |
| mmu_circRNA_013053 |
| mmu_circRNA_28460 |
| mmu_circRNA_29619 |
| mmu_circRNA_27163 |
| mmu_circRNA_33247 |
| mmu_circRNA_21857 |
| mmu_circRNA_30261 |
| mmu_circRNA_007066 |
| mmu_circRNA_40315 |
| mmu_circRNA_42255 |
| mmu_circRNA_33590 |
| mmu_circRNA_28910 |
| mmu_circRNA_39693 |
| mmu_circRNA_40823 |
| mmu_circRNA_43828 |
| mmu_circRNA_29836 |
| mmu_circRNA_25765 |
| mmu_circRNA_26154 |
| mmu_circRNA_45718 |
| mmu_circRNA_23387 |
| mmu_circRNA_21033 |
| mmu_circRNA_19555 |
| mmu_circRNA_44079 |
| mmu_circRNA_26049 |
| mmu_circRNA_41894 |
| mmu_circRNA_42123 |
| mmu_circRNA_23275 |
| mmu_circRNA_45805 |
| mmu_circRNA_32185 |
| mmu_circRNA_38243 |
| mmu_circRNA_40903 |
| mmu_circRNA_34109 |
| mmu_circRNA_31688 |
| mmu_circRNA_007853 |
| mmu_circRNA_19188 |
| mmu_circRNA_22496 |
| mmu_circRNA_35162 |
| mmu_circRNA_35588 |
| mmu_circRNA_33702 |
| mmu_circRNA_22278 |
| mmu_circRNA_006355 |
| mmu_circRNA_19499 |
| mmu_circRNA_40568 |
| mmu_circRNA_22197 |
| mmu_circRNA_27328 |
| mmu_circRNA_26697 |
| mmu_circRNA_21040 |
| mmu_circRNA_37848 |
| mmu_circRNA_45796 |
| mmu_circRNA_20332 |
| mmu_circRNA_19202 |
| mmu_circRNA_36551 |
| mmu_circRNA_005340 |
| mmu_circRNA_37381 |
| mmu_circRNA_34081 |
| mmu_circRNA_43882 |
| mmu_circRNA_26004 |
| mmu_circRNA_19761 |
| mmu_circRNA_22627 |
| mmu_circRNA_40244 |
| mmu_circRNA_017645 |
| mmu_circRNA_013120 |
| mmu_circRNA_012412 |
| mmu_circRNA_30664 |
| mmu_circRNA_19038 |
| mmu_circRNA_35956 |
| mmu_circRNA_38593 |
| mmu_circRNA_27344 |
| mmu_circRNA_012594 |
| mmu_circRNA_28640 |
| mmu_circRNA_19992 |
| mmu_circRNA_26502 |
| mmu_circRNA_001769 |
| mmu_circRNA_33996 |
| mmu_circRNA_22792 |
| mmu_circRNA_34515 |
| mmu_circRNA_40441 |
